# Effect of first-line and second-line selective laser trabeculoplasty on corneal hysteresis in patients with normal tension glaucoma: a multicenter study

**DOI:** 10.1007/s00417-024-06735-z

**Published:** 2025-01-06

**Authors:** Keigo Takagi, Koji Nitta, Maki Katai, Masaki Tanito

**Affiliations:** 1 Department of Ophthalmology, Shimane University Faculty of Medicine, Izumo, Japan; 2 Department of Ophthalmology, Fukui-ken Saiseikai Hospital, Fukui, Japan; 3 Department of Ophthalmology, NTT Medical Center Sapporo, Sapporo, Japan

## Abstract

**Key messages:**

*****What is known***:**

- Corneal hysteresis (CH) reflects the viscoelastic properties of the cornea.
- Glaucoma patients with low CH are at increased risk for visual field progression.
- The First-line or Second-line Selective Laser Trabeculoplasty (FSS) Study demonstrated that SLT is an effective and safe treatment for patients with normal tension glaucoma (NTG).

*****What is new***:**

- Early application of SLT in patients with lower intraocular pressure (IOP) is associated with an increase in CH, along with a significant reduction in IOP.

Corneal hysteresis (CH) is an indicator of the viscoelastic properties of the cornea, reflecting its ability to absorb and dissipate energy. It is measured using instruments such as the Ocular Response Analyzer (ORA, Reichert Technologies) or Corvis ST (OCULUS, Inc). Eyes with a more rigid cornea can be more susceptible to glaucomatous damage due to increased intraocular pressure (IOP). Thus, eyes with low CH are at higher risk for glaucoma progression [1]. The First-line or Second-line Selective Laser Trabeculoplasty (FSS) Study is a multicenter cohort study conducted at 26 centers in Japan to evaluate the effectiveness and safety of selective laser trabeculoplasty (SLT) in patients with normal tension glaucoma (NTG) [2]. Current study utilized data from the FSS Study to examine the impact of SLT on CH.

In this study, we included 93 eyes of 93 NTG patients from the FSS study who had their Goldmann-correlated intraocular pressure (IOPg), corneal-compensated intraocular pressure (IOPcc), and CH measured using ORA before and at 12 and 24 months after SLT. The calculation method involved using P1 as the inward applanation pressure and P2 as the outward applanation pressure when the air pulse hit the cornea, with IOPg as the average of these two values and CH as their difference (i.e., P1-P2). IOPcc was corrected by CH and calculated as P2–0.43*CH. Changes in each parameter from baseline at 12 and 24 months were analyzed using mixed-effects regression models for the overall, first-line, and second-line groups. The baseline characteristics of the current dataset are shown in Table 1. In the FSS study, the first-line group was defined as those receiving SLT as the initial IOP-lowering treatment for newly diagnosed NTG, while the second-line group included those receiving SLT after the use of one anti-glaucoma medication (prostaglandin analogs or β-blocker). Detailed definitions of disease and groups were described in our previous report [2].

**Table 1:** Baseline characteristics of participants

	All	First line	Second line	*p*-value
Eyes, n	93	54	39	
Age, years	61.8 ± 11.5	59.0 ± 10.9	65.8 ± 11.3	0.005
Eye laterality, right/left	45 (52)/48 (48)	25 (54)/29 (46)	20 (51)/19 (49)	0.68
Sex, male/female	33 (35)/60 (65)	21 (39)/33 (61)	12 (31)/27 (69)	0.51
Baseline IOP, mmHg	16.0 ± 2.5	16.1 ± 2.4	15.9 ± 2.6	0.77
MD, dB	−4.2 ± 4.0	−3.4 ± 3.8	−5.3 ± 4.0	0.03
CCT, µm	530 ± 31	531 ± 32	529 ± 30	0.87
ECD, cells /mm^2^	2645 ± 274	2665 ± 259	2617 ± 294	0.41

Data are expressed as mean ± standard deviation for continuous parameters and as N (%) for categorical parameters. P-values were calculated between the First-line and Second-line groups using the Student’s t-test for continuous parameters and Fisher’s exact probability test for categorical parameters. MD, visual field mean deviation; CCT, central corneal thickness; ECD, endothelial cell density

Goldmann applanation tonometry-measured IOP, and ORA-measured IOPg, IOPcc, and CH at baseline, 12, and 24 months are summarized in Table 2. In all participants, Goldmann IOP was significantly lower at both 12 and 24 months compared to the baseline value (*p* < 0.0001 for both). IOPg and IOPcc also decreased significantly at both 12 and 24 months from baseline (IOPg: *p* = 0.003 and *p* = 0.001; IOPcc: *p* = 0.003 and *p* = 0.02, respectively). Compared to baseline, a significant increase in CH was observed at 12 months (*p* = 0.01), but the difference was not significant at 24 months (*p* = 0.58). Subgroup analysis showed a significant increase in CH at 12 months in the first-line group (*p* = 0.01).

**Table 2:** Goldmann IOP, and ORA-measured IOPg, IOPcc, and CH before and after SLT

	All	*p*-value	First line	*p*-value	Second line	*p*-value
IOP-GAT, mmHg						
Baseline	16.0 ± 2.5	‑	16.1 ± 2.4	‑	15.9 ± 2.6	‑
12 months	12.8 ± 1.9	< 0.0001	12.7 ± 1.9	< 0.0001	13.0 ± 1.9	< 0.0001
24 months	12.9 ± 2.1	< 0.0001	12.9 ± 2.1	< 0.0001	13.0 ± 2.2	< 0.0001
IOPg, mmHg						
Baseline	16.5 ± 3.2	‑	16.7 ± 3.2	‑	16.2 ± 3.2	‑
12 months	14.4 ± 3.2	0.003	14.7 ± 3.4	0.02	14.1 ± 3.0	0.046
24 months	14.2 ± 3.2	0.001	14.8 ± 3.0	0.09	13.2 ± 3.2	0.002
IOPcc, mmHg						
Baseline	17.5 ± 4.2	‑	17.8 ± 4.9	‑	17.1 ± 3.0	‑
12 months	14.9 ± 2.7	0.003	15.0 ± 2.8	0.01	14.7 ± 2.6	0.048
24 months	15.0 ± 2.8	0.02	15.6 ± 2.8	0.28	14.3 ± 2.8	0.006
CH, mmHg						
Baseline	10.1 ± 1.3	‑	10.2 ± 1.2	‑	9.9 ± 1.4	‑
12 months	10.6 ± 1.2	0.01	10.7 ± 1.1	0.01	10.4 ± 1.4	0.26
24 months	10.3 ± 1.4	0.58	10.3 ± 1.1	0.72	10.2 ± 1.8	0.25

Data are expressed as mean ± standard deviation. For each parameter, p-values were calculated among Baseline, 12 months, and 24 months using a mixed-effects regression model. IOP-GAT, intraocular pressure measured by Goldmann applanation tonometry; IOPg, Goldmann-correlated IOP; IOPcc, corneal compensated IOP; CH, corneal hysteresis; ORA, Ocular Response Analyzer; SLT, selective laser trabeculoplasty

Additionally, using a multivariate mixed-effects regression model, a higher baseline IOPg was found to be a significant predictor of a larger increase in CH from baseline (estimate = 0.20/mmHg; *p* = 0.0003). In contrast, other parameters, including age (*p* = 0.63), sex (*p* = 0.053), first- or second-line treatment (*p* = 0.60), 12- or 24-months (*p* = 0.17), and changes in IOPg (*p* = 0.10), were not significantly associated.

Previous studies have shown an increase in CH following the initiation of topical prostaglandin therapy [3] or after trabeculectomy [4]. These treatments may enhance the cornea’s viscoelastic properties by reducing IOP, thereby decreasing stress on the optic nerve. In cases of open-angle glaucoma, both IOPg and IOPcc decreased, and CH increased after SLT with a minimal follow-up of 4 weeks [5]. In that study, the majority of eyes had high-pressure glaucoma, and SLT was applied after maximal use of topical medications (average medication score was 2.7) [5]. In recent years, there has been growing interest in using SLT from the early stages of treatment [6]. Our findings indicate that performing SLT in the early stages of treatment in patients with lower IOP also results in an increase in CH, accompanied by a significant reduction in IOP.

## Declarations

### The FSS Study group

Koji Nitta, Kae Sugihara, Akiko Narita, Tomoko Naito, Takako Miki, Maki Katai, Shiro Mizoue, Keiji Yoshikawa, Masaki Tanito, Kazuhisa Sugiyama, Yasushi Ikuno, Takuji Matsuda, Hiroaki Ozaki, Kazuyuki Hirooka, Kaori Komatsu, Yoshiaki Saito, Itaru Kimura, Tairo Kimura, Takeshi Sagara, Katsuyoshi Suzuki, Aika Tsutsui, Akiko Ishida, Toru Nakazawa, Satoru Tsuda, Toyoaki Tsumura, Naoki Tojo, Naoto Tokuda, Tadashi Nakano, Tomoyuki Watanabe, Kenji Nakamoto, Naka Shiratori, Mami Nanno, Naoya Nezu, Yoshitaka Tasaka, Shigeru Mori, Shigeki Yamabayashi, Kimihito Konno and Miyuki Domoto.
